# Gastrointestinal stromal tumors: a comprehensive radiological review

**DOI:** 10.1007/s11604-022-01305-x

**Published:** 2022-07-09

**Authors:** Akitoshi Inoue, Shinichi Ota, Michio Yamasaki, Bolorkhand Batsaikhan, Akira Furukawa, Yoshiyuki Watanabe

**Affiliations:** 1 Department of Radiology, Shiga University of Medical Science, Seta, Tsukinowa-cho, Otsu, Shiga 520-2192 Japan; 2 Department of Radiology, Mayo Clinic, Rochester, MN USA; 3 Department of Radiology, Nagahama Red Cross Hospital, Shiga, Japan; 4 Department of Radiology, Kohka Public Hospital, Shiga, Japan; 5 Graduate School of Human Health Sciences, Department of Radiological Science, Tokyo Metropolitan University, Tokyo, Japan

**Keywords:** Gastrointestinal stromal tumors, Acute abdomen, Proto-oncogene proteins c-kit, Multidetector computed tomography, Magnetic resonance imaging

## Abstract

Gastrointestinal stromal tumors (GISTs) originating from the interstitial cells of Cajal in the muscularis propria are the most common mesenchymal tumor of the gastrointestinal tract. Multiple modalities, including computed tomography (CT), magnetic resonance imaging (MRI), fluorodeoxyglucose positron emission tomography, ultrasonography, digital subtraction angiography, and endoscopy, have been performed to evaluate GISTs. CT is most frequently used for diagnosis, staging, surveillance, and response monitoring during molecularly targeted therapy in clinical practice. The diagnosis of GISTs is sometimes challenging because of the diverse imaging findings, such as anatomical location (esophagus, stomach, duodenum, small bowel, colorectum, appendix, and peritoneum), growth pattern, and enhancement pattern as well as the presence of necrosis, calcification, ulceration, early venous return, and metastasis. Imaging findings of GISTs treated with antineoplastic agents are quite different from those of other neoplasms (e.g. adenocarcinomas) because only subtle changes in size are seen even in responsive lesions. Furthermore, the recurrence pattern of GISTs is different from that of other neoplasms. This review discusses the advantages and disadvantages of each imaging modality, describes imaging findings obtained before and after treatment, presents a few cases of complicated GISTs, and discusses recent investigations performed using CT and MRI to predict histological risk grade, gene mutations, and patient outcomes.

## Introduction

Gastrointestinal stromal tumor (GIST) is the most common mesenchymal tumor of the gastrointestinal tract, which commonly occurs in middle-aged and elderly populations but infrequently in younger generations [1]. The prevalence of GISTs was estimated to be 15/100,000 people [2]. Imaging examinations fundamentally show GISTs as submucosal tumors of the gastrointestinal tract with various degrees of enhancement and reveal other findings. It is also known that the spectrum of imaging findings of GISTs is broad and the diagnosis of GISTs with uncommon imaging features is challenging. Furthermore, GISTs may lead to conditions, such as gastrointestinal hemorrhage, rupture, and bowel obstruction, that require urgent care. Imaging examinations, especially computed tomography (CT), play an important role in patient management. Because GISTs may arise in patients with a background of gene mutation, multiple GISTs imply the presence of gene mutations. Patients with multiple GISTs should undergo screening to detect accompanying tumors and be placed under appropriate surveillance. Recently, many articles have reported that sectional imaging using a radiomics approach is helpful in predicting histopathological grade, gene mutations, and patient outcomes [3–7].

Although several review articles regarding imaging have been published [8–16], in addition to describing well-known imaging findings of GISTs, this article updates the knowledge regarding imaging findings and research topics, and discusses the prospect of the importance of radiologists and imaging for physicians managing and treating patients with GISTs.

## Pathophysiology

**Fig. 1:**
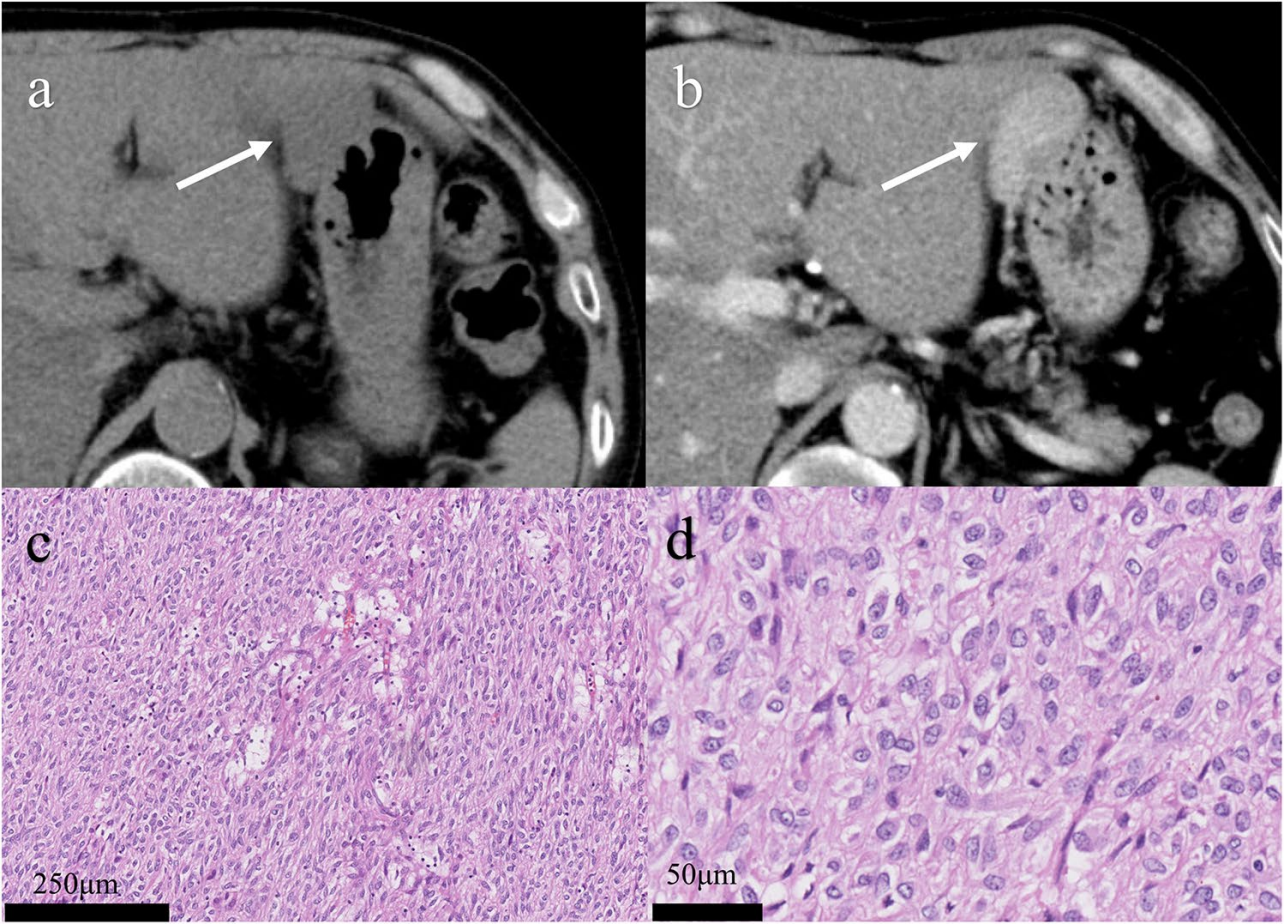
Low-risk gastrointestinal stromal tumor (epithelioid cell type). A 49-year-old male with a gastrointestinal stromal tumor arising from the stomach. An oval exophytic tumor arising from the stomach showing a slightly low density on noncontrast CT (**a**: arrow) and homogenous enhancement shown in the venous phase (**b**: arrow). Hematoxylin–eosin stain showing spindle cell-type tumors (**c**) but no mitosis in the high-power field (**d**)

**Fig. 2:**
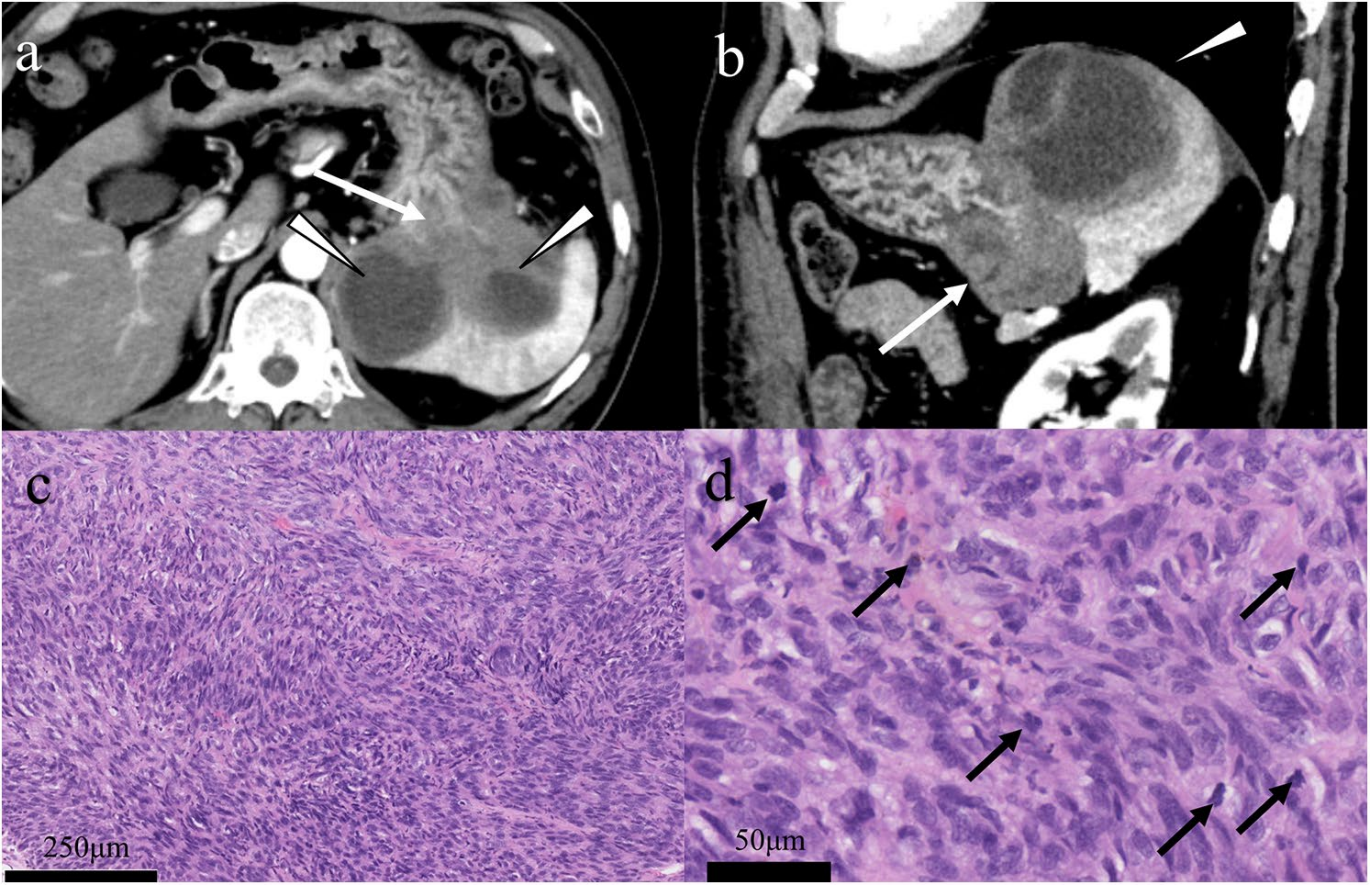
High-risk gastrointestinal stromal tumor (spindle cell type). A 65-year-old male with a gastrointestinal stromal tumor arising from the stomach. A lobulated tumor that originated from the stomach with exophytic growth includes a solid component (**a** and **b**: arrows) and cysts with thickened walls (**a** and **b**: arrowheads). The solid component shows moderate enhancement in the arterial phase continuing the gastric wall, and the cystic portion dislocates the spleen on multiplane CT images. Hematoxylin–eosin stain showing epithelioid-type tumor cells (**c**) and multiple mitoses in the high-power field (**d**: arrows)

GISTs arise from the interstitial cells of Cajal that are electrical pacemakers and mediators of enteric neurotransmission in the muscularis propria of the gastrointestinal tract [17]. GISTs can be located anywhere in the gastrointestinal tract and rarely in the peritoneum and retroperitoneal space. Histopathological evaluation is essential for both diagnosis and risk stratification in patients with GISTs. In hematoxylin–eosin staining, GISTs are morphologically classified as spindle cell type (70%), epithelioid cell type (20%), or mixed type [18]. The spindle and epithelioid cell types appear to correspond to leiomyomas and leiomyoblastomas in the classification before GISTs were defined [19]. The spindle cell type is long, skinny or fusiform shaped and the epithelioid cell type is round or polygonal (Figs. 1 and 2). Mixed-type GISTs include both spindle and epithelioid components. Depending on the morphology, immunohistochemistry, which is essential for distinguishing GISTs from other tumors, is performed. For this purpose, KIT, desmin, S100 protein, α-smooth muscle actin, CD34, discovered on GIST-1, signal transducer and activator of transcription 6, β-catenin, and anaplastic lymphoma kinase are used [20]. Ki-67 is useful for estimating the biological aggressiveness of GISTs. Succinate dehydrogenase B (SDHB) is necessary for GIST subtyping [21].

Risk classifications rely on tumor size, mitosis, and location and presence of a perforation. Fletcher et al. proposed the National Institution of Health (NIH) classification defined by tumor size and mitosis in 2002 (Table 1) [18]. Miettinen et al. advocated the Armed Forces Institute of Pathology (AFIP) classification defined by anatomical location, tumor size, and mitosis (Table 2) [22]. Joensuu et al. modified the NIH classification because tumor rupture during surgery was associated with poor prognosis. In their classification, ruptured tumors of any tumor size and mitotic count are categorized as high risk [23].

**Table 1:** National Institutes of Health classification

Risk	Timor size	Mitotic count
Very low	< 2 cm	< 5/50 HPF
Low	2–5 cm	< 5/50 HPF
Intermediate	< 5 cm	6–10/50 HPF
	5–10 cm	< 5/50 HPF
High	> 5 cm	> 5/50 HPF
	> 10 cm	Any mitotic rate
	Any size	> 10/50 HPF

*HPF* high-power field

**Table 2:** Armed Forces Institute of Pathology risk classification

Mitotic count	Tumor size	Stomach	Duodenum	Jejunum and ileum	Rectum
≤ 5/50 HPF	≤ 2 cm	None	None	None	None
	2.1–5 cm	Very low	Low	Low	Low
	5.1–10 cm	Low	Moderate	Insufficient data	Insufficient data
	> 10 cm	Moderate	High	High	High
> 5/50 HPF	≤ 2 cm	None	High	None	High
	2.1–5 cm	Moderate	High	High	High
	5.1–10 cm	High	High	Insufficient data	Insufficient data
	> 10 cm	High	High	High	High

*HPF* high-power field

## Imaging modality

Multiple modalities are used for sampling tissue, evaluating a tumor’s local extent, staging, predicting risk, conducting surveillance after surgery, monitoring response to molecularly targeted therapy, and sometimes for monitoring hemostasis only (e.g., endoscopy and digital subtraction angiography).

### Computed tomography

CT is the primary modality used for the initial diagnosis of GISTs, surgical planning, postsurgical surveillance, and monitoring therapy response owing to its ability to visualize exophytic and endophytic components and regional and distant metastases [24]. The density of GISTs on noncontrast CT is similar to that of the muscles and its enhancement varies. Intratumoral gas suggesting communication with the gastrointestinal lumen, calcification, and intratumoral hemorrhage are readily identified on CT, and CT enterography contributes to the identification of small-sized GISTs in the small bowel of patients with suspected small bowel bleeding [25]. The morphological features visualized on CT enable predicting high-grade GIST and poor prognosis [26–30]. In dual-energy spectral CT, a higher slope of the spectral curve and normalized iodine concentration indicate high-risk GISTs [31]. Recently, quite a few radiomics research groups have used CT images to estimate histopathological risk, gene mutation, or patient prognosis [32].

### Magnetic resonance imaging

On magnetic resonance imaging (MRI), GISTs typically show low signal intensity on T1-weighted imaging (T1WI), high signal intensity on T2WI, and enhanced signal intensity on post gadolinium images [33]. MRI generally provides morphological imaging findings similar to those obtained from CT; additionally, quantitative parameters, such as the apparent diffusion coefficient (ADC) and degree of enhancement, and perfusion parameters, are helpful in assessing malignancy and response to treatment, respectively [34–37]. MRI has advantages in diagnosing hepatic metastasis; a previous study reported that MRI detected additional hepatic metastasis, which was not detected via CT [38]. Additionally, MRI enables detailed visualization of the pelvic anatomy, including the anal sphincter and anal verge, as well as the tumor itself [39].

### Barium study

Upper gastrointestinal barium studies, small bowel follow-through, and barium enema were frequently performed in the past; however, now they are being replaced with endoscopy and sectional imaging examinations. GISTs are depicted as submucosal tumors with spherical ridges and normal mucosal surface when they grow endophytically [40]. This modality does not enable imaging the exterior of the gastrointestinal tract lumen (e.g., the whole image of exophytic GISTs, vasculature, degeneration, and metastasis).

### Ultrasonography

Ultrasonography is used for hepatic metastasis evaluation and in image-guided biopsy to obtain tissue for histopathological examination. Endoscopic ultrasonography (EUS) provides more detailed observation. Marginal lobulation possibly suggested malignant GISTs [41]. GISTs that are < 2 cm in diameter are typically homogeneous hypoechoic masses having a smooth margin arising from the fourth layer corresponding to the muscularis propria; however, differentiating GISTs from other submucosal tumors, such as leiomyomas, schwannomas, glomus tumors, and ectopic pancreas, is difficult because the EUS findings of GISTs and these tumors are similar [31]. c-kit staining performed as an additional examination using fine-needle aspiration is helpful to diagnose GISTs if the result is positive but insufficient if it is negative [42].

### Fluorodeoxyglucose positron emission tomography

Fluorodeoxyglucose positron emission tomography (FDG-PET) that enables visualization of viable tissue with glucose metabolism is helpful for distinguishing GISTs from non-GISTs and stratifying histopathological risk [43] as well as for performing initial disease evaluation and monitoring response to molecularly targeted therapy [44]. With regard to evaluating response to molecularly targeted therapy, FDG-PET/CT predicts near-term response for FDG-avid tumors with higher accuracy than CT [45]. Regardless of primary or metastatic lesions, GISTs consisting of viable neoplastic cells demonstrate FDG uptake. FDG-PET/CT shows a sensitivity of 89% and specificity of 97% for restaging and may result in changes in therapeutic strategy if it detects new positive or negative lesions [46]. However, false-positive lesions due to increased FDG uptake in inflammatory areas should be recognized, especially postoperatively [47].

### Digital subtraction angiography

GISTs in the small intestine are displayed as well-defined homogeneous hypervascular masses associated with early returning drainage veins. The obvious draining vein on the tumor surface is frequently seen even in small-sized GISTs (< 2 cm) of the small bowel [48]. Given the less invasive imaging examinations using CT and MRI and the possibility of endoscopic hemostasis, except in the small bowel, angiography is commonly performed to achieve hemostasis and treat tumoral bleeding caused by transarterial embolization in the small bowel.

### Endoscopy

GISTs are delineated as a bulging mass covered by the mucosa on endoscopy. The major advantage of endoscopy is tissue sampling that is essential for preoperative histopathological diagnosis. Another advantage is endoscopic hemostasis for GISTs with acute gastrointestinal bleeding. Furthermore, endoscopic observation of the small bowel requires the use of capsule endoscopy or double balloon endoscopy. However, capsule endoscopy requires a long interpretation time because of high number of images and the use of endoscopic procedures (i.e., biopsy and hemostasis) is not feasible and double balloon endoscopy is invasive, and needs both oral and anal approaches to examine the whole small bowel.

## Imaging findings

As GISTs arise from the myenteric plexus in the muscularis propria, they commonly appear as submucosal tumors, which are defined as intramural growth underneath the mucosa, in the findings of any modality or pathology, regardless of its location. However, imaging findings of GISTs are diverse because they have a broad range of locations, growth patterns (endophytic, intramural, and exophytic), and enhancement patterns (hypervascular, intermediate, and delayed enhancement patterns). Additionally, some GISTs are related to necrosis, calcification, ulceration, drainage vein, and regional/distant metastasis. However, these imaging findings tend to show small GISTs (< 5 cm) as round and homogeneous tumors and large GISTs (≥ 5 cm) as lobulated and heterogeneous tumors, and they are frequently related to intratumoral degenerations, including necrosis, calcification, ulceration, and metastasis [34].

### Location

The most common location of primary lesions is the stomach (60%), followed by the small intestine (30%), duodenum (5%), colon (4%), and esophagus/appendix (1%) [49]. Esophageal GISTs are located predominantly in the distal portion of the esophagus [50]. In the duodenum, the second portion of the duodenum is the most common site [51]. High-risk GISTs more frequently occur in the ileum than in the duodenum and jejunum [52]. GISTs potentially arise from any part of the gastrointestinal tract and are reported as GISTs derived from Meckel’s diverticulum [53]. Additionally, GISTs arising outside the gastrointestinal tract are referred to as extragastrointestinal GISTs (E-GISTs). At the time of detection, E-GISTs are larger (mean diameter: 15.6 cm) in the greater omentum, mesentery, and retroperitoneum [54].

### Growth pattern

Generally, the growth pattern of GISTs is categorized as exophytic, intraluminal, and mixed/combined/endophytic. In gastric GISTs, despite no deviation in the growth pattern, exophytic and mixed growth patterns are correlated with high-grade GISTs and high mitotic counts [55]. Exophytic (54%) and mixed growth patterns (39%) are common in the small bowel [56], and mixed growth pattern is dominant in the duodenum (76.5%) [51]. The growth pattern of GISTs probably depends on the surrounding anatomy.

### Enhancement pattern

**Fig. 3:**
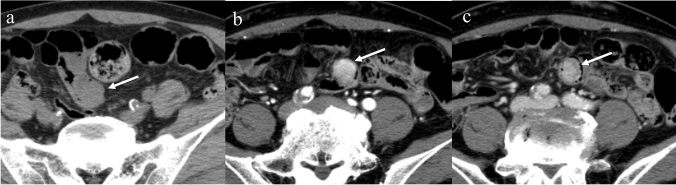
Enhancement of a small gastrointestinal stromal tumor in the small bowel. A 70-year-old male with a small gastrointestinal stromal tumor in the small bowel. An endophytic round mass is observed (**a**–**c**: arrows). The mass shows marked enhancement in the arterial (**b**: arrow) and venous phases (**c**: arrow) and washout patterns

**Fig. 4:**
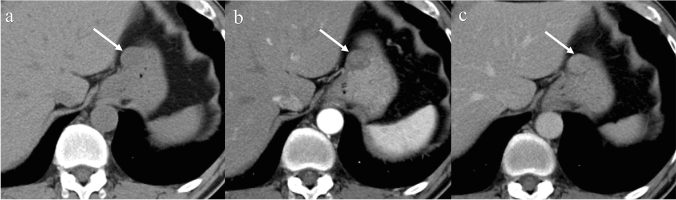
Enhancement of a small gastrointestinal stromal tumor in the stomach. A 49-year-old male with a small gastrointestinal stromal tumor in the stomach. An intramural round circumscribed mass is seen in the anterior gastric wall (**a**–**c**: arrows). The mass shows mild enhancement in the arterial phase (**b**: arrow) and enhancement equivalent to that of the gastric wall in the venous phase (**c**: arrow)

The contrast enhancement of GISTs has been described as homogeneous moderate contrast (in small GISTs) and heterogeneous enhancement [57, 58]; however, the enhancement degree and pattern differ between gastric and small bowel GISTs. With regard to small-sized GISTs (< 5 cm), small bowel GISTs show a washout pattern with marked enhancement during arterial phase (Fig. 3), whereas gastric GISTs show a plateau pattern with intermediate enhancement (Figs. 4). Even in large-sized GISTs (≥ 5 cm), the arterial phase reveals a significant difference in contrast enhancement [59]. The enhancement degree in the venous phase gradually increases from the duodenum to the ileum [52]. Small bowel GISTs and neuroendocrine neoplasms show a hypervascular pattern, adenocarcinomas and lymphomas show a delayed enhancement pattern, and metastatic tumors displays an intermediate enhancement pattern [60]. In a comparison of GISTs vs. non-GISTs, small intestine GISTs showed a higher degree of contrast enhancement than non-GISTs, including metastatic tumors, lymphomas, neuroendocrine neoplasms, desmoids, and schwannoma [61].

### Early venous return

**Fig. 5:**
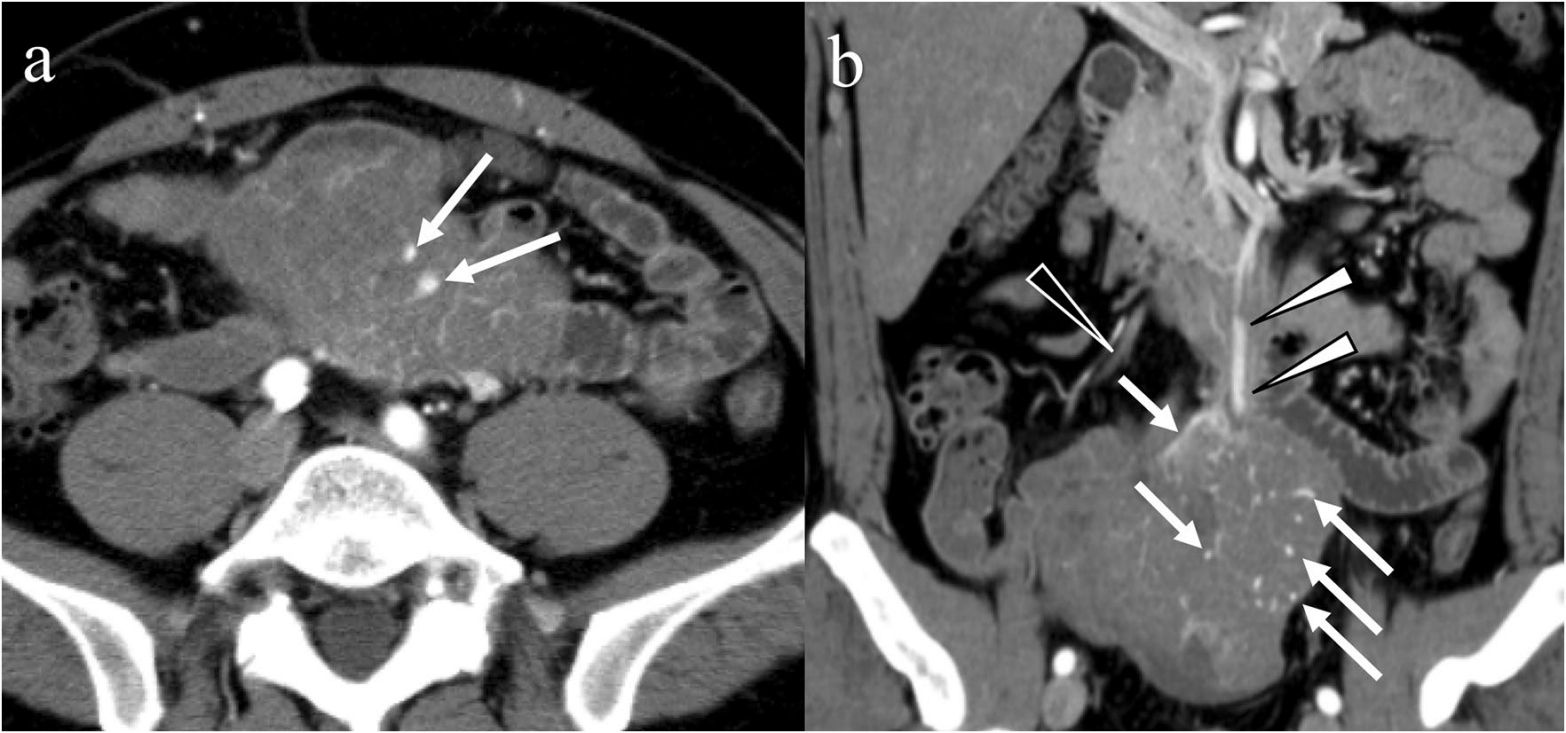
Early venous return. A 46-year-old male with an exophytic gastrointestinal stromal tumor of the small bowel. The tumor contains enlarged vasculature in both the central and peripheral areas (**a**, **b**: arrows). Although the ileocecal vein is not enhanced (**b**: black arrowhead), a dilated vein shows contrast enhancement suggesting early venous return from the small bowel mass in the arterial phase (**b**: white arrowhead)

Tumor vessel sign, which is defined as the presence of conspicuous vessels that can be directly traced from the tumor margin to the named vessels, is helpful for identifying the origin of hypervascular tumors [62]. As early development of a draining vein is frequently observed in small bowel GISTs on digital subtraction angiography [48, 63], early venous return, which is defined as the return of contrast media in the arterial phase, associated with an enlarged draining vein is common in small bowel GISTs (Fig. 5) but not in gastric GISTs [59]. The diameter of the draining vein is positively correlated with the tumor size. Large GISTs in size may mimic retroperitoneal, gynecological, or inguinal neoplasms [64]. In sectional imaging, tracing a vein showing early venous return provides a clue to the identity of the tumor’s origin site; however, early venous return is also observed in hypervascular tumors, such as small bowel metastasis of renal cell carcinoma [61].

### Other findings

**Fig. 6:**
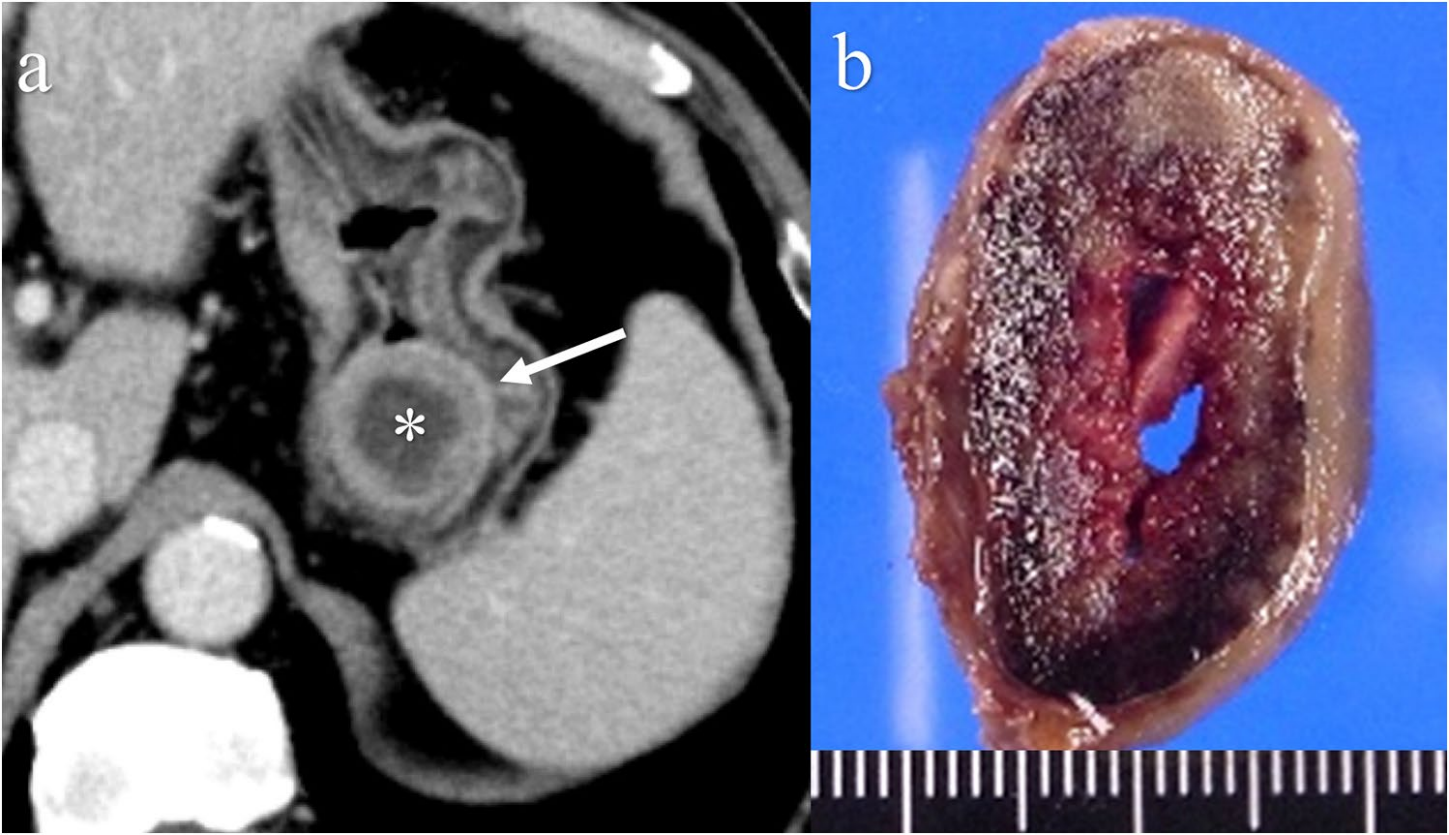
Cystic degeneration of a gastrointestinal stromal tumor in the stomach. A 66-year-old male with a submucosal tumor in the stomach detected via upper gastrointestinal endoscopy. Contrast-enhanced CT showing an endophytic tumor (**a**: arrow) and fluid collection (**a**: asterisk). A tumor consisting of a red solid component with a cavity at the center on a surgical specimen (**b**)

Necrosis that is described as a cystic component on ultrasonography, CT, and MRI is observed in 39% of small bowel GISTs (Fig. 6) [56]. Limited to small bowel GISTs (≥ 5 cm), necrosis is seen in 66.7–100% of these tumors, and intratumoral hemorrhage, which is defined as a hyperdense area on noncontrast CT, is seen in 66.7–88.9% of these tumors [59]. Intratumoral hemorrhage or necrosis develops when the hypervascular tumor outgrows its blood supply, which is associated with a heterogeneous tumor texture.

**Fig. 7:**
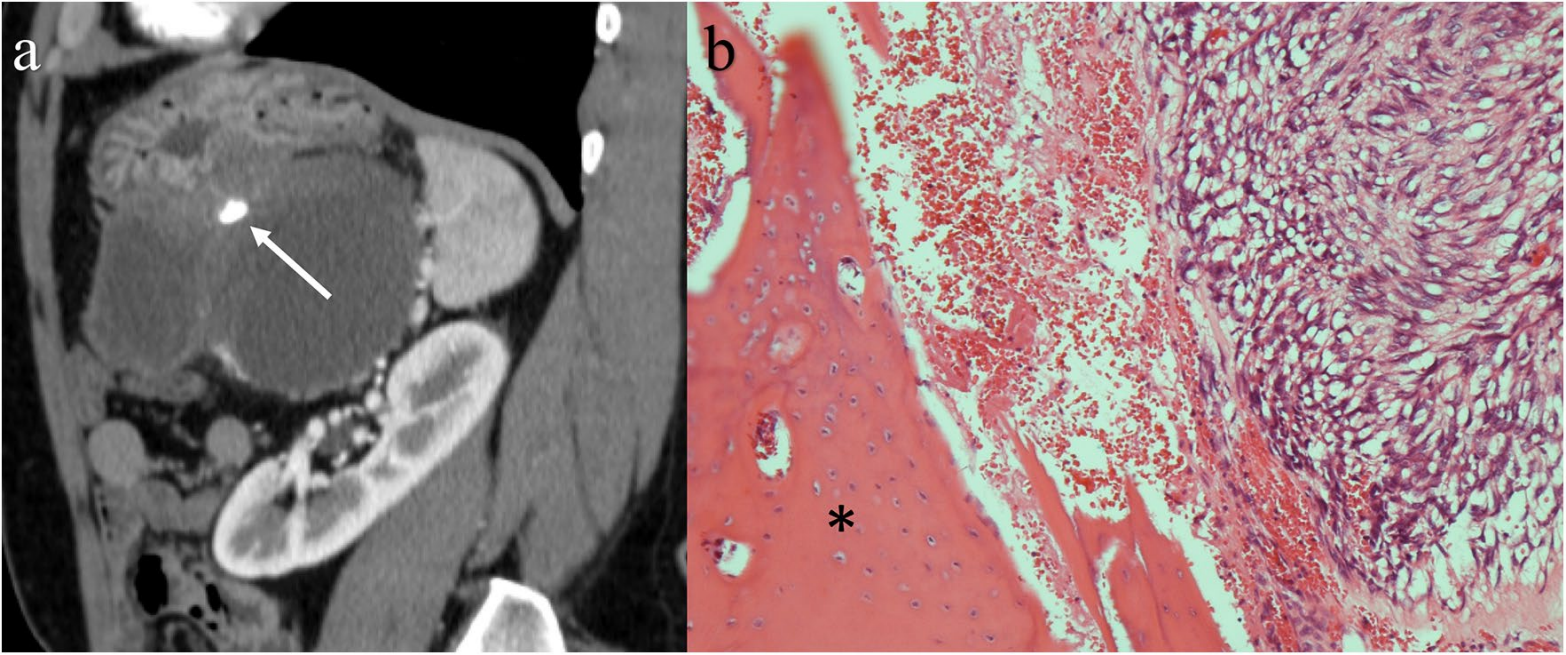
Calcification in a gastrointestinal stromal tumor in the stomach. A 52-year-old male with a cystic mass in his left-upper abdomen detected via ultrasonography during an annual health check-up. Contrast-enhanced CT showing a solid tumor arising from the gastric wall associated with cystic degeneration. Calcification in the solid component can be seen (**a**: arrow). Bone tissue in the solid component is shown in a surgical specimen (hematoxylin–eosin stain: × 100) (**b**: asterisk)

Calcification is seen in 7–22% of small bowel GISTs [56, 59]. Calcification forming a stippled-to-coarsely granular appearance is seen in 10.5% of GISTs on histopathology and is occasionally associated with metaplastic bones (Fig. 7). Although calcification is more common in large GISTs and potentially associated with high risk [58], calcification itself is not a significant predictor of prognosis in histopathological analysis [22].

GISTs, regardless of the size, are commonly associated with ulceration that results in gastrointestinal bleeding, and in large tumors, it results in cavitation/fistula, rupture, and perforation. On sectional imaging, ulceration and cavitation/fistula are seen as a recess on the surface of the tumor and tract continuing to the lumen that contains gas or fluid, respectively. They are seen as pooling barium with various depth degrees and shapes on fluoroscopy. Ulceration or cavitation is frequently associated with reactive lymphadenopathy [61].

### Metastasis

**Fig. 8:**
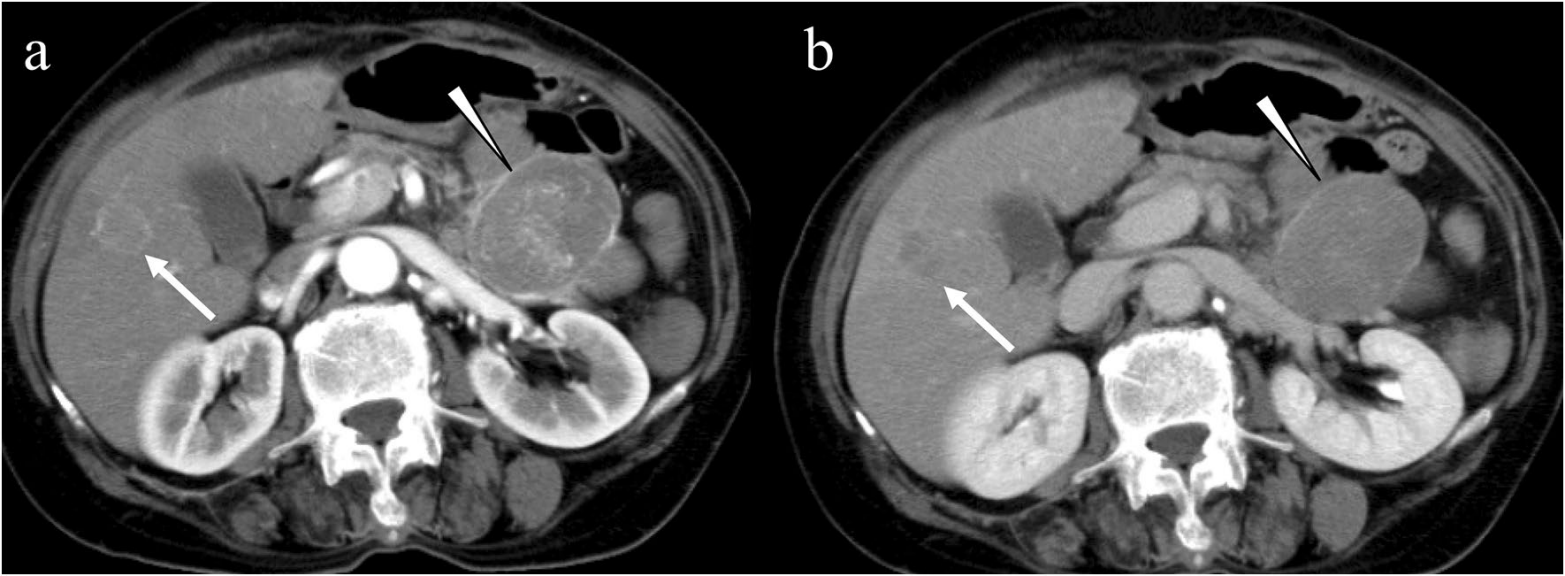
Hepatic metastasis of a gastrointestinal stromal tumor in the small bowel. A 69-yar-old female with a gastrointestinal stromal tumor in the small bowel and hepatic metastasis. A slightly hyperenhanced mass is seen in the liver in the arterial phase (**a**: arrow) and shows washout in the portal venous phase (**b**: arrow). Bulky masses in the small bowel are seen (**a** and **b**: arrowheads)

**Fig. 9:**
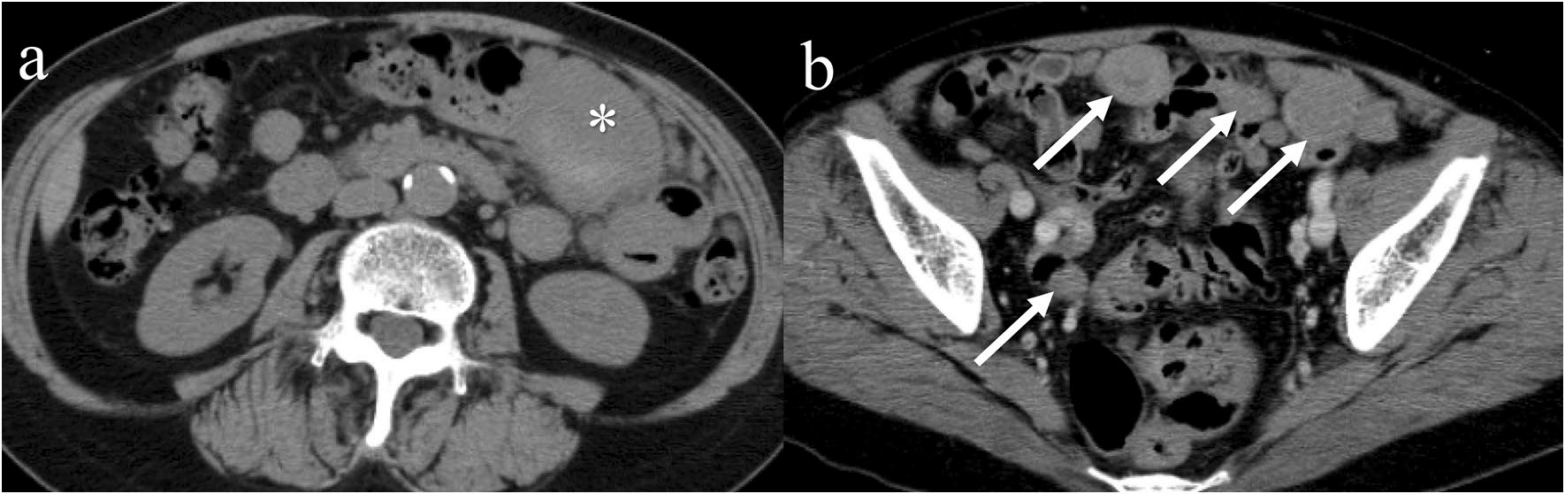
Peritoneal metastasis of a gastrointestinal stromal tumor in the small bowel. A 67-year-old female with a gastrointestinal stromal tumor in the small bowel presenting with peritoneal sarcomatosis. On noncontrast CT, the primary lesion is seen as a high-attenuation area, indicating intratumoral hemorrhage (**a**: asterisk). Contrast-enhanced CT showing multiple round masses in the greater omentum adjacent to the bowels, suggesting serosal implants (**b**: arrows)

GISTs mainly metastasize to the liver and peritoneum and rarely to the retroperitoneum, lymph nodes, lung, ovaries, kidneys, gallbladder, pleura, subcutaneous tissue, bone, and brain [38, 65–67]. Unlike adenocarcinoma, only 6% of patients with GISTs are diagnosed with nodal metastasis during the follow-up period [68]. However, in pediatric or adult pediatric-type GISTs, lymph node metastasis is common (20–59%) [1]. Lung metastasis is reported in 10.1% of patients with GISTs and 98.4% of those have bulky abdominal metastasis (i.e., liver and/or peritoneal metastasis); therefore, thoracic imaging can be reserved for those patients [69]. In a dynamic study, most liver metastases were hypervascular in the arterial phase, showed washout patterns in the venous phase (Fig. 8) [13, 38]. Similar to primary lesions, large metastases are frequently associated with necrosis and fluid collection. Peritoneal dissemination of GISTs is known as peritoneal sarcomatosis, which is an entity analogous to peritoneal carcinomatosis and mainly caused by adenocarcinoma. Peritoneal sarcomatosis often shows spherical and hypervascular implants but neither ascites nor obstruction of hollow organs (Fig. 9), whereas peritoneal carcinomatosis tends to be flat or ovoid and causes bowel obstruction and hydronephrosis [70]. Hemoperitoneum and acute-onset symptoms, such as abdominal pain and hypovolemic shock, indicate ruptured liver or peritoneal metastasis.

## Imaging findings of recurrence after molecularly targeted therapy

GISTs ≥ 2 cm in size should be resected completely, and smaller GISTs (< 2 cm) may be endoscopically monitored every 6–12 months. CT is the primary imaging modality used for surveillance after surgery and for monitoring response during molecularly targeted therapy. Patients with completely resected GISTs are regularly followed up depending on the risk category. The GIST Guideline Subcommittee in Japan recommends CT to be performed every 6–12 months for very low-, low-, and intermediate-risk GISTs and every 4–6 months for high-risk GISTs [71]. For children and young adults, MRI, contrast-enhanced ultrasonography, and FDG-PET are preferred modalities instead of CT [72].

**Fig. 10:**
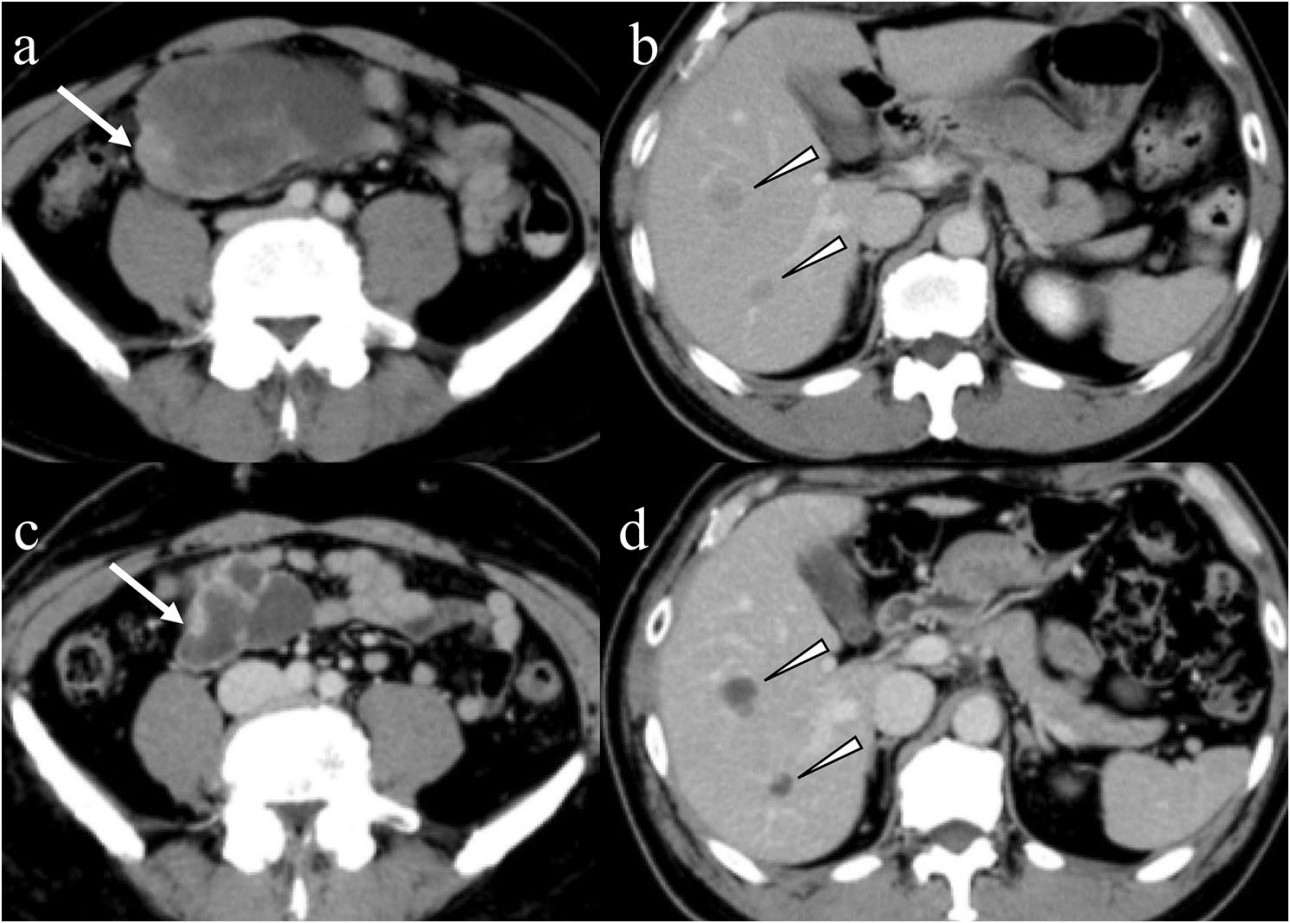
Imaging findings after imatinib therapy. A 54-year-old male with high-risk gastrointestinal stromal tumor in the small bowel was treated with imatinib. A bulky tumor with degeneration (**a**: arrows) and hepatic metastases (**b**: arrowheads) are seen in the venous phase of contrast-enhanced CT. The primary small bowel lesion decreased in size (**c**: arrow). Hepatic metastases show no change in size, whereas the contrast enhancement markedly decreased (**d**: arrowheads)

Outcomes of patients with high-risk, metastatic, or recurrent GISTs dramatically improved after administering imatinib, a tyrosine kinase inhibitor (TKI), as an adjuvant therapy. At present, several multikinase inhibitors, such as sunitinib and regorafenib, are being clinically used for the treatment of GISTs resistant to imatinib [73]. Since response to molecularly targeted therapy is visualized only as a minor volume reduction even in cases showing response (Fig. 10), the Response Evaluation Criteria in Solid Tumors (RECIST) categorized tumor response into four categories (complete response, partial response, stable disease, and progressive disease) focusing only on tumor diameter as a limitation in monitoring GISTs [74]. Choi et al. developed new CT response criteria for GISTs treated with imatinib and defined a > 10% decrease in tumor size or > 15% decrease in tumor density as partial response (Table 3) [75]. However, the Choi criteria still have some issues because the occurrence of intratumoral hemorrhage and cystic or myxoid degeneration mimicking disease progression are frequent during TKI therapy [24]. Furthermore, the nodule-within-mass pattern is common and reported as an important sign of recurrent GISTs [76], and peripheral thickening in cystic degeneration is related to progression during imatinib therapy [77]; however, these imaging findings suggest that recurrence does not affect the category in both the RECIST and Choi criteria. Intratumoral iodine quantification in dual-energy CT, which is not influenced by hemorrhage and calcification unlike CT number, demonstrated superiority in assessing response during TKI therapy according to the RECIST 1.1 and modified Choi criteria [78].

**Table 3:** Summary of the Choi and RECIST 1.1 criteria

	Choi criteria (75)	RECIST 1.1 (74)
CR	Disappearance of all lesions No new lesions	Disappearance of all lesions, all nodal lesions have a short axis < 10 mm No new lesions
PR	A ≥ 10% decrease in size or a ≥ 15 decrease in tumor density HU No new lesion No obvious progression of nonmeasurable disease	≥ 30% decrease in the sum of the target-lesion diameters No new lesion No progression of nontarget lesions
SD	Does not match the other criteria	Does not match the other criteria
PD	A ≥ 10% increase in tumor size and does not match criteria of PR by tumor density New lesions New intratumoral nodules or increase in the size of the existing intratumoral nodules	≥ 20% increase in the sum of diameters as referenced to an absolute increase of ≥ 5 mm

*RECIST* Response evaluation criteria in solid tumors, *CR* complete response, *PR* partial response, *SD*, stable disease, *PD*, progressive disease

FDG-PET is more sensitive than morphological imaging in evaluating therapy response [24]. Two major response criteria, the European Organization for Research and Treatment of Cancer criteria using the maximum standardized uptake value [79] and the PET response criteria in solid tumors criteria employing the peak of the standardized uptake value corrected for lean body mass [(Lean body mass) = (Total weight) – (Fat mass)] [80] have been disseminated (Table 4). These criteria include four categories (complete metabolic response, partial metabolic response, stable disease, and progressive metabolic disease) that correspond to the categories in the RECIST criteria [81]. The advantage of FDG-PET imaging is that it detects responding tumors earlier than size measurement in morphological imaging [82]. Similarly, with regard to qualitative MRI parameters, increasing ADC at 1 week after imatinib therapy can predict a good response to imatinib therapy [36].

**Table 4:** Summary of the EORTC and PERCIST 1.0 criteria

	EORTC (79)	PERCIST 1.0 (80)
CMR	Complete resolution of FDG uptake in all lesions	Complete resolution of FDG uptake in all lesions
PMR	A > 25% reduction in the sum of SUVmax after more than one cycle of therapy	A ≥ 30% reduction in SULpeak and an absolute drop of 0.8 SULpeak units
SMD	Does not qualify for other categories	Does not qualify for other categories
PMD	A > 25% increase in the sum of SUVmax New FDG-avid lesions	A ≥ 30% increase in SULpeak and absolute increase of 0.8 SULpeak units New FDG-avid lesion

*EORTC* European organization for research and treatment of cancer, *PERCIST* PET response criteria in solid tumors, *CMR* complete metabolic response, *PMR* progressive metabolic disease, *PMR* partial metabolic response, *SMD* stable metabolic disease, *FDG* fluorodeoxyglucose, *SUVmax* maximum standardized uptake value, *SULpeak* peak lean body mass standardized uptake value

## Syndromic gastrointestinal stromal tumors

*KIT* mutation is seen in most GISTs and other genotypes, such as platelet-derived growth factor receptor-alpha (PDGFRA), succinate dehydrogenase (SDH), and neurofibromin gene mutations, are related to neoplastic cell proliferation in GISTs. Most GISTs that are commonly associated with KIT and PDGFRA sporadically occur in middle-to-older-age populations (> 40 years old).

**Fig. 11:**
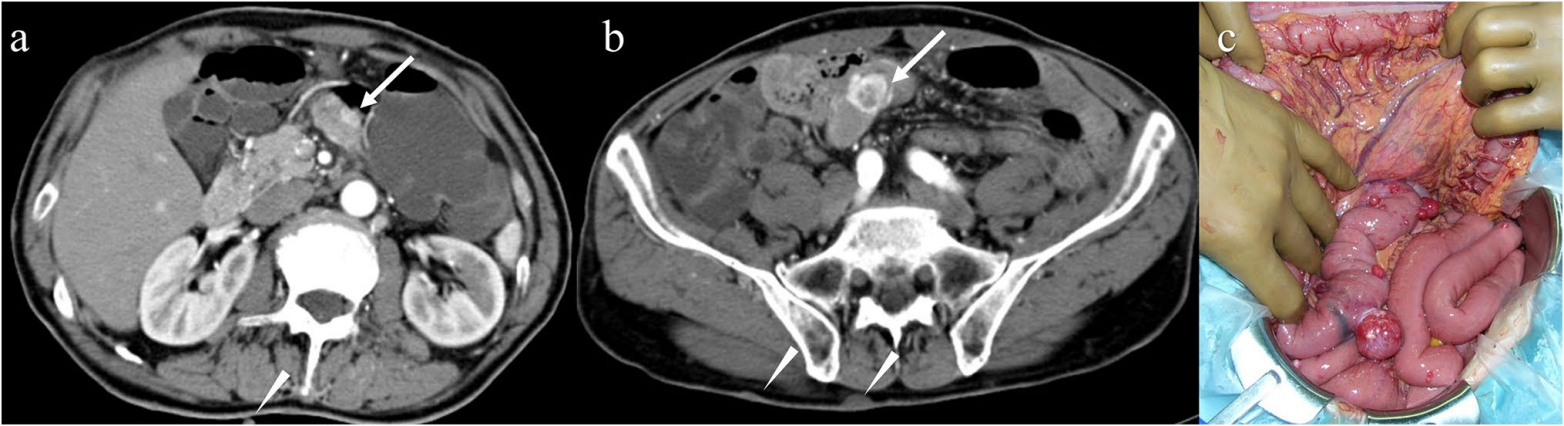
Multiple gastrointestinal stromal tumors associated with neurofibromatosis type 1. A 70-year-old male presented with melena. His hemoglobin level decreased from 14.0 g/dL to 6.0 g/dL within 1 month. He was diagnosed with neurofibromatosis type 1. Axial contrast-enhanced CT images showing multiple hypervascular tumors of various sizes in the small bowel (**a**, **b**: arrows). Note that multiple skin lesions are compatible with neurofibromas (**a**, **b**: arrowheads). Laparotomy reveals multiple exophytic nodules in the small intestine (**c**)

**Fig. 12:**
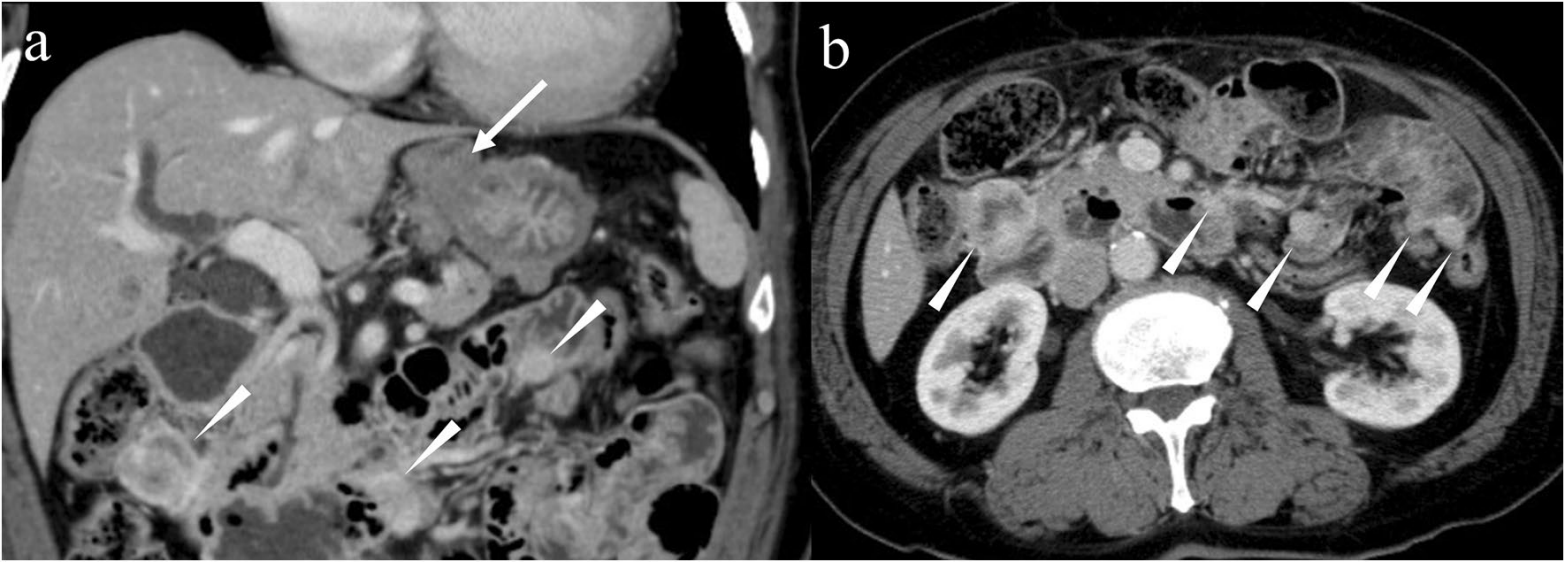
Multiple gastrointestinal stromal tumors associated with a familial gastrointestinal stromal tumor. A 78-year-old male presented with anemia. Coronal and axial contrast-enhanced CT in the venous phase demonstrate a homogeneous hypovascular tumor in the stomach (**a**: arrow) and multiple homogeneous or heterogeneous hypervascular tumors in the duodenum and jejunum (**a** and **b**: arrowheads). Diverse imaging findings (i.e., hypovascular vs. hypervascular and homogeneous vs. heterogeneous) are observed even in the same patient

In patients with multiple GISTs, related syndromes should be considered. Approximately 7% of patients with neurofibromatosis type 1 (NF-1) have multiple GISTs exclusively in the small intestine (Fig. 11). The lack of function of neurofibromin leads to the constitutive activation of the RAS oncogene and the subsequent activation of the RAS/RAF/MAP kinase pathway [83]. On CT, multiple round tumors are seen in the small bowel and skin lesions suggesting neurofibroma are diagnostic clues for NF-1-related GISTs. Another disease entity of multiple GISTs is familial GIST caused by *KIT* or *PDGFRA* mutation transferred via autosomal dominant inheritance. Familial GISTs are distributed in the stomach, small bowel, and colon (Fig. 12), unlike NF-1-related GISTs. Hyperpigmentation characterized by KIT activation is common in familial GISTs [21].

In pediatric and adult pediatric-type GISTs (genetically similar to pediatric GIST and seen in adults), deficiency of a subunit of SDH (SDHB) may play a pathological role. Dysfunction of SDH results in the accumulation of substrate succinate, leading to stabilization of HIF-1α that then activates the transcription of target oncogenes [21]. Additionally, the Carney triad is characterized by multiple gastric GISTs, paragangliomas, and pulmonary chondromas with female predominance, and Carney–Stratakis syndrome that involves multiple gastric GISTs and paragangliomas is associated with SDHB dysfunction [84].

## Complications

Patients with large GISTs may complain of acute symptoms [11]. Palliative care may be administered to manage symptoms of complicated GISTs prior to surgery, although complete surgical resection is the standard therapy if resectable [85].

**Fig. 13:**
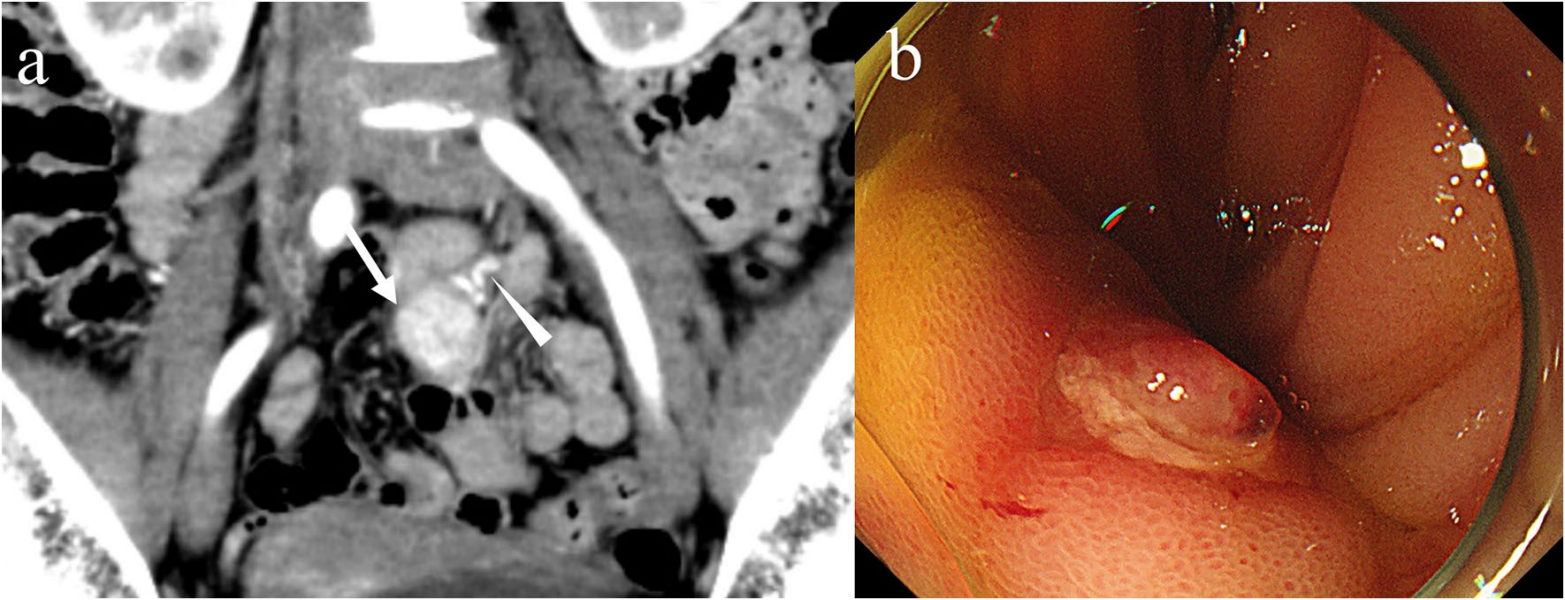
Gastrointestinal bleeding caused by a gastrointestinal stromal tumor in the small bowel. A 59-year-old female visited an emergency department with a continuous tarry stool after hematochezia. Her hemoglobin level decreased from 12.0 g/dL to 8.3 g/dl for a day. Contrast-enhanced CT showing a hypervascular tumor in the small intestine (**a**: arrow). The enlarged vein continuing to the tumor is markedly enhanced in the arterial phase (**a**: arrowhead). Double balloon endoscopy showing a submucosal tumor with ulcer (**b**). Inoue A. Gastrointestinal stromal tumor (GIST). (In Mizunuma K, et al., eds. *Diagnosis Imaging and Intervention Therapy of Abdominal Emergencies*. Tokyo: MedicalView; 2018. P200, Fig. 1

Gastrointestinal bleeding is the most common complication of GISTs because they are frequently associated with mucosal ulceration [86]. Clinical manifestation (i.e., hematemesis, hematochezia, and melena) depends on the location and degree of bleeding. Extravasation of iodine contrast material, indicating active arterial bleeding from a corresponding point, and hyperattenuating intraluminal material, suggesting recent hemorrhage, are critical findings of gastrointestinal bleeding on CT [87]. Extravasation of iodine contrast media is frequently observed in patients with acute massive gastrointestinal bleeding presenting hemodynamical instability [88]. However, gastrointestinal bleeding caused by GISTs is generally not massive [89]. Therefore, in patients presenting with gastrointestinal bleeding, it is reasonable to regard the tumor as the cause if it is found on CT (Fig. 13). Endoscopic hemostasis is a common hemostatic approach for the management of upper gastrointestinal bleeding caused by GISTs [31]. For small bowel bleeding, double balloon enteroscopy can be used to stop the bleeding; however, it is not suitable for an urgent situation [90]. For all locations, transarterial embolization is an effective approach to control gastrointestinal bleeding caused by GISTs [91].

**Fig. 14:**
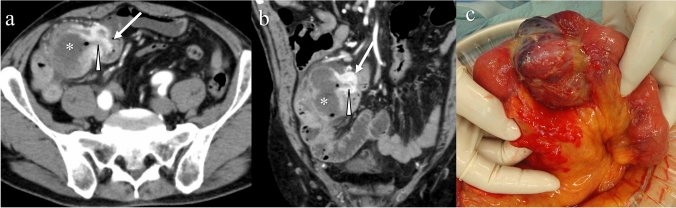
Ruptured gastrointestinal stromal tumor in the small bowel. A 72-year-old male presented with epigastralgia and his pain gradually migrated from the upper abdomen to the right lower abdomen, resembling a symptom of acute appendicitis. Axial (**a**) and coronal (**b**) images of contrast-enhanced CT in the arterial phase showing the hypervascular tumor (arrows) in the small bowel and adjacent fluid collection (asterisks) with fistula (arrowheads). Laparotomy showing that the tumor ruptured into the mesentery (**c**)

Large GISTs have been associated with rupture resulting in abscess, hemoperitoneum, or perforation. A common symptom of ruptured GISTs is abdominal pain, and patients may present with other systemic or gastrointestinal symptoms [92]. Ruptured GISTs are associated with an heterogeneous texture, laminated or whirled appearance, and hemoperitoneum on ultrasonography and CT [93]. Large size, large eccentric necrosis, wall defects, and lobulated shape are common appearances of ruptured GISTs on CT (Fig. 14) [94]. Pneumoperitoneum is associated with perforated GISTs wherein communication exists between the gastrointestinal tract and abdominal cavity [95]. Hemoperitoneum is more common than pneumoperitoneum because GISTs are hypervascular and grow exophytically without communication with the gastrointestinal lumen [94].

**Fig. 15:**
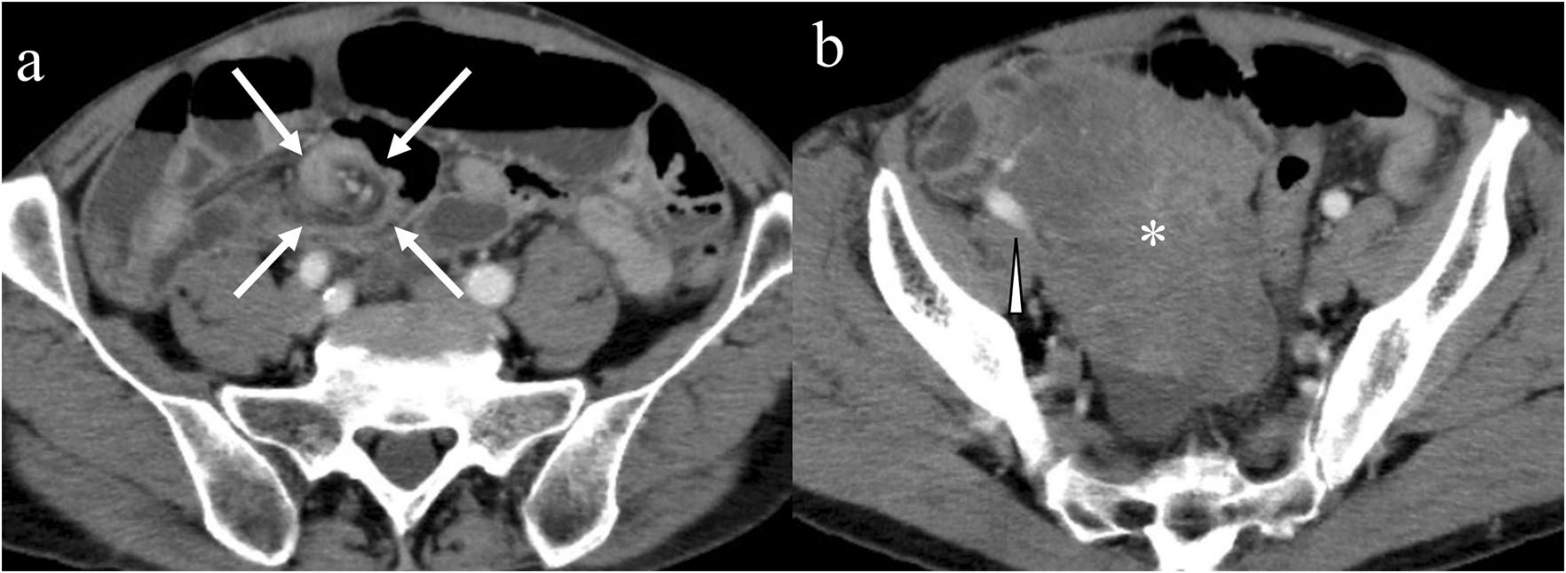
Mesenteric volvulus caused by an exophytic gastrointestinal stromal tumor in the small intestine. A 74-year-old male presented with abdominal discomfort and bloating. Contrast-enhanced CT shows the whirl sign in the mesentery, but enhancement is preserved in the involved mesenteric vessels (**a**: arrows). A > 10 cm tumor is seen in the pelvis (**b**: asterisk). The dilated drainage vein with enhancement is observed adjacent to the tumor (**b**: arrowhead). Laparotomy showing a large exophytic lobulated tumor arising from the small bowel on the antimesenteric side

GISTs may cause small bowel obstruction via three mechanisms influenced by their growth patterns [86]. First, GISTs growing endophytically may evoke intussusception by acting as an intussusceptum [96]. Second, GISTs that narrow the intestinal lumen may cause bowel obstruction. Third, large GISTs with an exophytic growth pattern may cause torsion involving the small bowel and mesentery (Fig. 15). Pedunculated exophytic GISTs may present with isolated tumor torsion [97].

## Risk prediction via sectional imaging

Visual qualitative assessment is simple and can be easily performed in clinical practice; however, intraobserver or interobserver reliabilities are limitations. Since tumor size is included in the NIH and AFIP criteria, it is obvious that tumor size on sectional imaging is also correlated with high-grade GISTs. As expected, hepatic and peritoneal metastases and mesenteric invasion are relevant to high-grade GISTs too. The presence of necrosis and ulceration, irregular shape, heterogeneous density, enlarged vessels, and exophytic/mixed growth pattern are related to high-grade GISTs (26–30). Chang et al. developed a CT-based nomogram including the aforementioned imaging findings predictive of the malignancy of gastric GISTs [98]. An irregular tumor shape and exophytic or mixed growth pattern are correlated with high mitotic counts in gastric GISTs (≥ 2 cm), which allows prediction of high mitotic counts [55]. For the prediction of the Ki-67 index, necrosis and tumor size are important imaging findings [99].

Radiomics is another approach to the task that has the benefit of using quantitative data beyond human interpretation; however, a large number of patients or lesions is required to obtain precise results. Similar to the limitations of visual assessments, limitations related to the intraobserver or interobserver variability exists in the lesion segmentation [32]. The AFIP classification considers taking anatomical locations into consideration for risk classification [22]. Furthermore, CT findings, especially enhancement features, differ considerably with anatomical locations [52, 59]. A few studies have focused on GISTs of the small bowel only [3, 4]; however, most studies employing a radiomics approach have included GISTs of any location to predict histopathological risk [29], gene mutation (*KIT* exon 11 mutation that is likely to respond to imatinib therapy) (5), Ki-67 index [6], or prognosis [7]. Development of radiomics requires a large number of cases, but the heterogeneity of tumor location is a major limitation. The radiomics approach can be extended by classifying GISTs on the basis of anatomical location, which may offer better performance in the future.

Recently, machine learning has been employed in the investigational research of GISTs. Wang et al. reported that a machine learning model distinguished gastric schwannomas from GISTs with excellent accuracy (area under the curve: 0.97) [100]. Yang et al. developed a binary prediction model for mitotic count (area under the curve: 0.80) [101]. Furthermore, Kang et al. reported a CT-based deep-learning model that effectively predicted histological risk (low, intermediate, and high risks) with an accuracy of > 0.77 [102].

## Summary

GISTs are the most common mesenchymal tumor of the gastrointestinal tract. Although imaging findings show a fundamental submucosal appearance of GISTs, a variety of imaging findings are known. In addition to the purposes of diagnosis, staging, surveillance, and symptomatic GIST evaluation, imaging examinations, especially CT, are used to monitor responses to molecularly targeted therapy. Volume reduction of GISTs is subtle even in cases of response to therapy, unlike that of other neoplasms. Classic qualitative and novel radiomics approaches are being investigated as predictive tools for histological risk, gene mutations, and patient outcomes.
